# Reducing Implant Infection in Orthopaedics (RIIiO): a pilot study for a randomised controlled trial comparing the influence of forced air versus resistive fabric warming technologies on postoperative infection rates following orthopaedic implant surgery in adults

**DOI:** 10.1186/s13063-018-3011-y

**Published:** 2018-11-19

**Authors:** Michelle Kümin, Christopher Mark Harper, Mike Reed, Stephen Bremner, Nicky Perry, Matthew Scarborough

**Affiliations:** 1 Nuffield Department of Medicine, University of Oxford, Oxford, UK; 2 Brighton and Sussex University Hospitals NHS Trust, Brighton, UK; 3 Northumbria Healthcare NHS Foundation Trust, Hexham, UK; 4 Brighton and Sussex Medical School, Brighton, UK; 5 Oxford University Hospitals NHS Foundation Trust, Oxford, UK

**Keywords:** Surgical site infection, intraoperative hypothermia, forced air warming, resistive fabric warming, hemiarthroplasty

## Abstract

**Background:**

Approximately 70,000 to 75,000 proximal femoral fracture repairs take place in the UK each year. Hemiarthroplasty is the preferred treatment for adults aged over 60 years. Postoperative infection affects up to 3% of patients and is the single most common reason for early return to theatre. Ultraclean ventilation was introduced to help mitigate the risk of infection, but it may also contribute to inadvertent perioperative hypothermia, which itself is a risk for postoperative infection. To counter this, active intraoperative warming is used for all procedures that take 30 min or more. Forced air warming (FAW) and resistive fabric warming (RFW) are the two principal techniques used for this purpose; they are equally effective in prevention of inadvertent perioperative hypothermia, but it is not known which is associated with the lowest infection rates. Deep surgical site infection doubles operative costs, triples investigation costs and quadruples ward costs. The Reducing Implant Infection in Orthopaedics (RIIiO) study seeks to compare infection rates with FAW versus RFW after hemiarthroplasty for hip fracture. A cost-neutral intervention capable of reducing postoperative infection rates would likely lead to a change in practice, yield significant savings for the health economy, reduce overall exposure to antibiotics and improve outcomes following hip fracture in the elderly. The findings may be transferable to other orthopaedic implant procedures and to non-orthopaedic surgical specialties.

**Methods:**

RIIiO is a parallel group, open label study randomising hip fracture patients over 60 years of age who are undergoing hemiarthroplasty to RFW or FAW. Participants are followed up for 3 months. Definitive deep surgical site infection within 90 days of surgery, the primary endpoint, is determined by a blinded endpoint committee.

**Discussion:**

Hemiarthroplasty carries a risk of deep surgical site infection of approximately 3%. In order to provide 90% power to demonstrate an absolute risk reduction of 1%, using a 5% significance level, a full trial would need to recruit approximately 8630 participants. A pilot study is being conducted in the first instance to demonstrate that recruitment and data management strategies are appropriate and robust before embarking on a large multi-centre trial.

**Trial registration:**

ISRCTN, ISRCTN74612906. Registered on 27 February 2017.

**Electronic supplementary material:**

The online version of this article (10.1186/s13063-018-3011-y) contains supplementary material, which is available to authorized users.

## Background

Hip fracture is a common problem globally and a major cause of hospitalisation in the elderly. Approximately 70,000 to 75,000 proximal femoral fracture repairs take place in the UK each year, with the number continuing to rise with the ageing population. Effective treatment requires early surgery, most commonly with hemiarthroplasty. Postoperative infection remains one of the most serious complications of this procedure, affecting 2.5–3.5% of patients, and is the single most common reason for early return to theatre [1]. Additionally, length of hospitalisation is doubled, further surgery is often required and the cost of treatment is substantial [2]. Elderly patients who have to undergo re-operation are often left with impaired mobility and are unable to return to independent living. Furthermore, mortality after an infective complication is generally three times higher than that following uncomplicated surgery [3].

There are several factors that influence the risk of postoperative wound infection, including comorbidities, preoperative waiting time and the duration of surgery. Ultraclean ventilation in the operating theatre was introduced to limit the rates of infection. It is most commonly delivered through laminar flow canopies, which are now used in more than 60% of hospitals in the UK [4]. Nevertheless, a major drawback of laminar air flow ventilation [5] is that it makes the patient colder than conventional ventilation, with inadvertent perioperative hypothermia (IPH) being itself a known risk factor for infection. Following the demonstration that patient warming reduces the rate of surgical site infections (SSIs) in colorectal surgery [6], both the National Institute for Heath and Care Excellence (NICE) and the World Health Organization recommended maintenance of normothermia with active warming devices for all operations lasting longer than 30 min [7, 8].

Numerous intraoperative warming methods exist [9]. Forced air warming (FAW) – or convective air-warming transfer – has traditionally been considered the most effective non-invasive method of transferring heat to the patient, with systematic reviews conducted a decade apart showing that FAW is still the dominant technique in use [10, 11]. Resistive fabric warming (RFW), an air-free method of warming patients that works on a similar principle to an electric blanket, thus using conduction rather than convection, was included as an option for perioperative warming in recent NICE guidelines [12]. A systematic review of 67 randomised controlled studies from 1964 to October 2015 was not able to recommend one technique over the other for the prevention of IPH [11], yet their influence on postoperative infection rate is unknown.

Mobilisation of non-sterile air at floor level by FAW could potentially be compromising the sterility of the surgical site [13, 14]. Additionally, despite FAW filtration systems meeting HEPA standards, potentially pathogenic organisms have been found in hoses and blower systems [15–19]. Avidan et al. [16] found that higher airborne bacterial loads were associated with higher infection rates in patients kept warm with FAW, but this was not confirmed in later studies [13, 20, 21] and has been actively challenged by others [22–24]. Therefore, negating the protective effects of laminar airflow is highly disputed since the evidence does not directly link disruption of laminar airflow ventilation by FAW with risk of infection [25–29]. Until more is known about the potential influence of FAW on the incidence of SSIs, recent recommendations to not install laminar airflow in operating rooms for the purpose of preventing SSIs should not be implemented [30, 31].

Deep SSIs double operative costs, triple investigation costs and quadruple ward costs [2]. A cost-neutral intervention capable of reducing postoperative infection rates would likely lead to a change in practice, yield significant savings for the health economy and reduce overall exposure to antibiotics, as well as improve outcomes for hip fractures in the elderly. The RIIiO study compares infection rates with FAW or RFW after hemiarthroplasty for hip fracture. The findings may be transferable to other non-orthopaedic surgical specialties.

## Methods/Design

### Study hypothesis and objectives

**Fig. 1:**
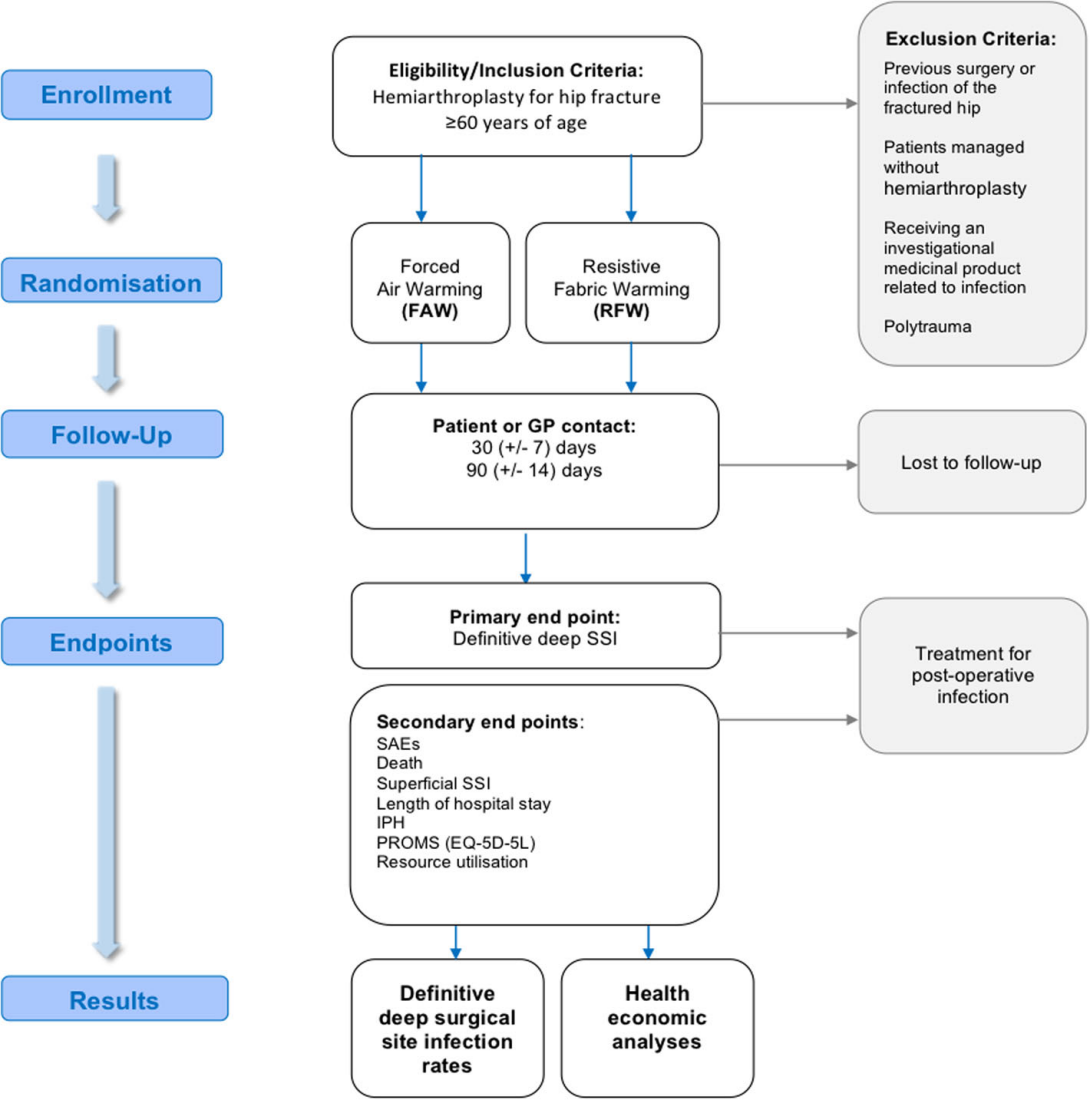
Flowchart of the participant pathway in the RIIiO pilot study

**Fig. 2:**
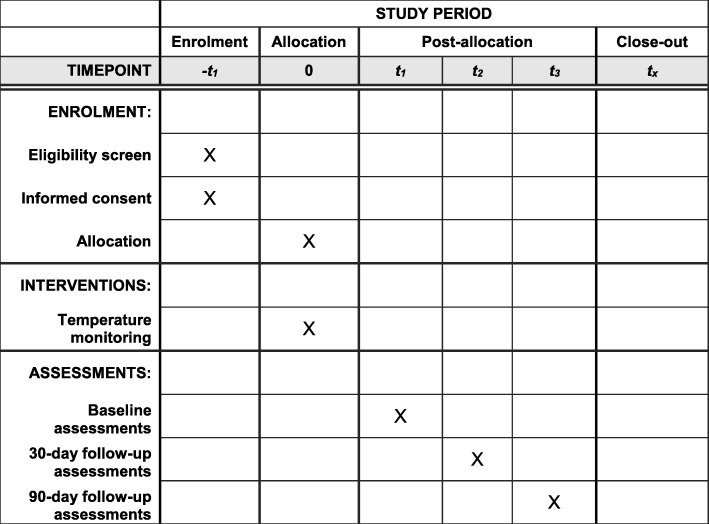
SPIRIT figure for the RIIiO pilot study

We postulate that the risk of postoperative orthopaedic implant infection may be influenced by the choice of intraoperative warming technology used to prevent IPH during hemiarthroplasty for hip fracture in elderly patients. RIIiO is a multicentre, parallel group, open label study randomising adults aged 60 years or over undergoing hemiarthroplasty following hip fracture to RFW or FAW. The primary endpoint is the observed event rate for definitive deep SSI within 90 days as determined by a blinded endpoint committee. A pilot study is being conducted in the first instance to inform the recruitment and data management strategies for the full trial. The pathway for participants is shown in Fig. 1 and summarised in the SPIRIT figure in Fig. 2 and SPIRIT checklist in Additional file 1.

### Trial participants and informed consent

Potential participants are identified from admission records, theatre lists and from daily trauma meetings at six NHS hospitals in the UK. Determination of eligibility is based on a review of the case notes and a clinical assessment in relation to the inclusion and exclusion criteria. Patients with hip fracture are a surgical priority and will normally undergo surgery on the next available operating list. Such patients have a high incidence of comorbidities, will inevitably have suffered trauma and are likely to either be in pain or to have received opiate analgesia. In this emergency setting, it is inappropriate and not always possible to ask potential participants to review trial documentation. Given the number of factors influencing capacity or the ability to communicate an informed opinion, those patients who are listed for surgery on the next available operating list are not approached for consent prior to their surgery. Preoperative consent for randomisation is sought from an appropriate consultee in accordance with section 32, subsection 9b of the 2005 Mental Capacity Act in the UK. At the earliest opportunity after recovery from surgery, randomised participants are provided with the study information (Additional file 2) and written personal consent to continue in the pilot study is sought. For any participant who continues to lack capacity, postoperative written agreement from a personal consultee is sought. In all cases, consent is received by appropriately qualified and Good Clinical Practice-trained research staff. Participants and their consultees are given the option to withdraw from the study at any time.

### Inclusion and exclusion criteria

Participants must meet all of the following criteria:

(1) Provision of informed consent OR consultee declaration, (2) aged 60 years or over, (3) presenting with fracture of the hip and (4) scheduled to undergo hemiarthroplasty.

Patients may not enter the study if any of the following apply:

(1) Previous surgery or infection of the affected hip, (2) hip fractures related to polytrauma, (3) patients managed without hemiarthroplasty or (4) receiving an investigational medicinal product related to infection. Polytrauma is defined as ‘multiple severe injuries involving three or more parts of the body’.

### Randomisation

Prior to surgery, and after confirmation of a patient’s eligibility, participants are allocated using simple randomisation, 1:1, in randomly permuted blocks of varying size without stratification by centre. Randomisation is through an established software package (MACRO) to either FAW or direct contact RFW. In the case of software failure, randomisation envelopes prepared in advance under the supervision of a qualified statistician are available to the local study team for immediate use in the emergency setting. The local research teams are responsible for randomisation and for informing the patient’s general practitioner that they are participating in the study.

### Study intervention

Only patients that undergo hemiarthroplasty can be recruited to this study. During their surgery, the participant is kept warm as part of their standard care using the technology to which they have been assigned. Both FAW and RFW are licenced and established techniques and are equally effective at preventing IPH [32–34]. Both warming devices are used in accordance with national guidelines as defined in NICE CG65. Temperature is measured just before the induction of anaesthesia, every 30 min during surgery, at the end of surgery and upon arrival in the recovery room. All thermometers are calibrated according to the standard protocol at each site. Whenever possible, temperature is measured with the automated ‘SpotOn zfd’ temperature monitoring system. IPH is defined as a temperature of less than 36 °C at the end of surgery or upon arrival in the recovery room. Where necessary for optimal clinical care, additional warming methods, such as actively warming intravenous fluids and blood products, may be employed at the discretion of the supervising clinician.

### Baseline assessments

The following data are recorded at baseline, the majority being captured from routine clinical care records: (1) age, (2) sex, (3) estimated height and weight, (4) American Society of Anaesthesiologists physical status classification, (5) anatomical side affected, (6) date of admission, (7) date of surgery, (8) randomisation arm, (9) adherence to randomisation result, (10) duration of surgery, (11) use of ultraclean ventilation in theatre, (12) cemented or uncemented prosthesis, (13) type of antibiotic-containing cement, (14) antimicrobial prophylaxis, (15) immuno-suppressants, (16) comorbidities (active malignancy, history of ischaemic heart disease, peripheral vascular disease, stroke, dementia, kidney disease/renal failure, diabetes mellitus, rheumatoid arthritis, systemic autoimmune disease and HIV), and (17) quality of life measures. Quality of life measures are obtained through the EQ-5D-5L questionnaire, which has been recommended for patients with hip fracture [35]. For those participants who lack capacity, the EQ-5D-5L questionnaire is completed by proxy through a consultee [36].

### Subsequent assessments

Subsequent assessments are undertaken at 30 (± 7) days and 90 (± 14) days after surgery. In addition to recording EQ-5D-5L, the patients’ medical records are consulted for any indication of deep or superficial SSI. Follow-up data include (1) date of discharge, (2) duration of hospital stay, (3) date[s] of readmission[s], (4) date of diagnosis of a potential deep SSI, (5) whether repeat surgery was performed, (6) radiological evidence of deep infection, (7) symptoms and signs indicative of a potential deep SSI (i.e. temperature, localised pain or tenderness, deep purulence from the wound or periprosthetic drain, spontaneous deep wound dehiscence), (8) results of deep tissue samples taken for histological analysis, (9) confirmed presence of microorganisms cultured from deep tissue/fluid samples (*Staphylococcus aureus*, coagulase-negative Staphylococcus sp., Streptococcus sp., Enterococcus sp., Pseudomonas sp. and/or other Gram negative organism[s]), (10) superficial SSI (i.e. involving only skin and subcutaneous tissues, purulent drainage from superficial incision, wound deliberately opened by the medical team due to pain or tenderness, erythema, localised swelling and/or warmth, positive aseptically obtained specimen from superficial incision or subcutaneous tissues (*S. aureus*, coagulase-negative Staphylococcus sp., Streptococcus sp., Enterococcus sp., Pseudomonas sp. and/or other Gram negative organism[s]), or (11) serious adverse events, including death (i.e. all-cause mortality).

### Definitions of deep and superficial SSI

Deep and superficial SSI definitions are adapted from the Centres for Disease Control SSI criteria published in January 2016 [37]. Deep SSI is defined by the following criteria:

1. Infection arising within 90 days of the index surgery (where day 1 is the procedure date) AND
2. Involves deep tissues related to the incision (e.g. fascial and muscle layers, joint space or periprosthetic region) AND
3. At least one of the following:
  i. Purulent drainage from the deep incision or periprosthetic drain
  ii. A deep incision that spontaneously dehisces, or is deliberately opened or aspirated or biopsied by a surgeon, physician or other designee and an organism is identified by a culture- or non-culture-based microbiologic testing method performed for purposes of clinical diagnosis or treatment (e.g. not Active Surveillance Culture/Testing), or without a culture- or non-culture-based microbiologic testing method being performed
  iii. An abscess or other evidence of infection involving the deep incision or periprosthetic region that is detected on gross anatomical, histopathological exam or imaging test

Superficial SSI is defined by the following criteria:

- Infection arising within 30 days of the index surgery (where day 1 is the procedure date) AND
- Involves only skin or subcutaneous tissue related to the incision AND
- At least one of the following:
  i. Purulent drainage from the superficial incision
  ii. Organisms identified from an aseptically obtained specimen from the superficial incision or subcutaneous tissue by a culture- or non-culture-based microbiologic testing method which is performed for purposes of clinical diagnosis or treatment (e.g. not Active Surveillance Culture/Testing (ASC/AST)
  iii. Superficial incision that is deliberately opened by a surgeon, physician or other designee and culture- or non-culture-based testing is not performed AND
  iv. The patient has at least one of the following signs or symptoms: pain or tenderness, localised swelling, erythema, heat
  v. Diagnosis of a superficial incisional SSI by a surgeon or physician

### Endpoints and limitation of bias

The primary endpoint of the pilot study is the observed event rate for definitive deep SSI within 90 days of surgery. Secondary endpoints of the study are (1) superficial SSI, (2) IPH, (3) length of hospital stay, (4) EQ-5D-5L measures, (5) resource utilisation and (6) serious adverse events, including death. Any post-randomisation readmission, clinic attendance or return to theatre with signs and symptoms at the site of surgery is considered a potential primary endpoint. Double blinding is not possible in this study. Any consequent risk of bias is limited by the use of a blinded endpoint committee (EPC) comprising clinicians with expertise in the diagnosis and management of bone and joint infection. For all potential deep SSIs, the EPC is provided with a summary of the participant’s medical records relevant to the clinical episode redacted for personal identifiers and any information relating to their randomisation or intraoperative thermoregulation. The EPC confirms if a primary endpoint has been reached. Superficial SSIs are identified by the local care team.

### The number of participants

Hemiarthroplasty carries a risk of deep SSI of approximately 2.5–3.5%. In order to provide 90% power to demonstrate an absolute risk reduction of 1%, using a 5% significance level, the full trial will need to recruit approximately 8630 participants over a 3-year period from a total of 30 sites (sample size calculations were performed in Stata, version 14SE [StataCorp, College Station, Texas]). The primary objective of the pilot study is to demonstrate that strategies for recruitment and data management for a trial of this size are appropriate and robust. Thus, there is no defined upper limit for the number of participants that can be recruited to the pilot study. Participants are recruited over a minimum period of 12 months at each site. To be able to keep the number of centres involved in the full trial to a maximum of 30, each pilot centre will be expected to recruit an average of two participants per week in the pilot study. The total number of patients recruited in the pilot study will be used to confirm the actual number of sites required for the full trial.

### Trial management and safety reporting

The pilot study is co-ordinated by the Brighton and Sussex Clinical Trials Unit and a trial management group. A trial steering committee (TSC), comprising patient and public representatives, two independent clinicians and a statistician, makes recommendations to the trial management group regarding the conduct of the trial, recruitment and follow-up rates, and assesses the progression plan to the full trial based on extrapolation of data acquired in the pilot study. An independent Data Safety Monitoring Board (DSMB) evaluates patient safety and frequency of endpoints in an un-blinded analysis and makes recommendations to the TSC.

## Discussion

A systematic review of 67 RCTs involving patient warming systems from 1964 to October 2015 could not identify whether FAW or RFW was more efficient at warming the patient [11]. To accurately study IPH and its consequences for orthopaedic patients, including postoperative infection rate, a standardised temperature monitoring protocol in a prospective trial with robust follow-up and adherence to CONSORT standards is needed [38]. An observational study in one hospital over a 2.5-year period suggested that the risk of developing deep infection up to 60 days after surgery was substantially greater for patients treated with FAW than RFW [14], but there were significant confounding factors in this study. The traditional assumption that FAW is the most effective non-invasive method of transferring heat to the body is based on warming comparisons that did not include RFW. Moreover, reduced infection rates with FAW have only been demonstrated, to date, with colorectal surgery [6], which is significantly different to orthopaedic trauma surgery. The RIIiO study will make substantial advances in the scientific understanding of whether or not the choice of patient warming technology influences the incidence of deep SSI. The pilot study will inform recruitment and data management strategies for such a trial.

### Trial status

The RIIiO pilot study began recruiting on April 3, 2017, in Northumbria Healthcare NHS Foundation Trust and subsequently in Brighton and Sussex University Hospitals NHS Trust, Oxford University Hospitals NHS Foundation Trust, Milton Keynes University Hospital NHS Foundation Trust, Heart of England NHS Foundation Trust, Sheffield  Teaching Hospitals NHS Foundation Trust and East Kent Hospitals University NHS Foundation Trust. Recruitment will cease in September 2018 and the pilot study will end in December 2018.

## Additional files

Additional file 1: RIIiO pilot study SPIRIT checklist. (DOC 122 kb)

Additional file 2: RIIiO pilot generic PIS consent form. RIIiO pilot study generic patient information sheet and consent form: the information provided to patients and their consultees containing the form used to record their consent for enrolment in the trial. (PDF 529 kb)

Additional file 3: Full ethically approved RIIiO pilot study protocol. (PDF 1940 kb)

## Abbreviations

EPC: Endpoint committee
FAW: forced air warming
GCP: good clinical practice
IPH: inadvertent perioperative hypothermia
NHS: National Health Service
NICE: National Institute for Health and Care Excellence
RFW: resistive fabric warming
SSI: surgical site infection
TSC/DSMB: Trial Steering Committee / Data Safety Monitoring Committee
