# Supplementary material for: Reducing Implant Infection in Orthopaedics (RIIiO): a pilot study for a randomised controlled trial comparing the influence of forced air versus resistive fabric warming technologies on postoperative infection rates following orthopaedic implant surgery in adults
Source: Trials. 2018 Nov 19;19:640. doi: 10.1186/s13063-018-3011-y (PMC6245696; doi:10.1186/s13063-018-3011-y)
Supplement: Supplementary file 2 — RIIiO pilot generic PIS consent form. RIIiO pilot study generic patient information sheet and consent form: the information provided to patients and their consultees containing the form used to record their consent for enrolment in the trial. (PDF 529 kb) [file 13063_2018_3011_MOESM2_ESM.pdf]

[Site to insert details]

Reducing Implant Infection in Orthopaedics

RIIiO

Pilot Study

Study Information Sheet &

Participant/Consultee

Consent/Advice form

IRAS number: 197521

RIIiO Pilot Study

This booklet explains what the RIIiO Pilot Study is all about, why it is being done and what it involves for those patients who participate. Please take time to read this information and discuss it with others if you wish. If there is anything that is not clear, or if you would like more information, please ask us.

What is the purpose of this study?

Unfortunately, some patients develop an infection after surgical repair for a hip fracture. These infections can be treated but often involve further surgery, a course of antibiotics and a lengthy recovery period. This is disruptive for patients, expensive for the NHS and promotes the emergence of antibiotic resistance. We know that keeping patients warm during hip operations reduces the risk of infection. Many patients are not actually aware that they are warmed during surgery or that different methods of keeping patients warm are used in different hospitals. The purpose of this study is to compare two warming methods, called 'Forced Air Warming' and 'direct contact Resistive Fabric Warming', to see if one method is associated with fewer post-operative infections than the other. If there is a difference, this will help us to improve patient care and make significant savings for the NHS.

Both methods are currently being used in NHS hospitals and both methods are equally good at keeping patients warm. One method may be better than the other, however, at helping to reduce the risk of infection.

A pilot study in a small number of hospitals is being conducted in the first instance to show that it will be possible to recruit enough patients for a full trial across the UK.

What is Forced Air Warming?

Forced Air Warming (FAW) uses an electrical heater and a fan to blow warm air through a hollow paper duvet placed over the patient. There are holes in the duvet for the warm air to come out and heat the patient like a hair dryer. At the moment, most hospitals use this system.

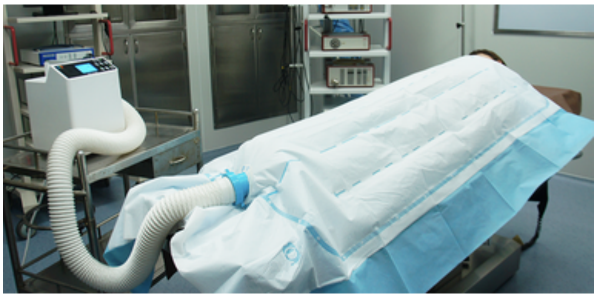

What is direct contact Resistive Fabric Warming?

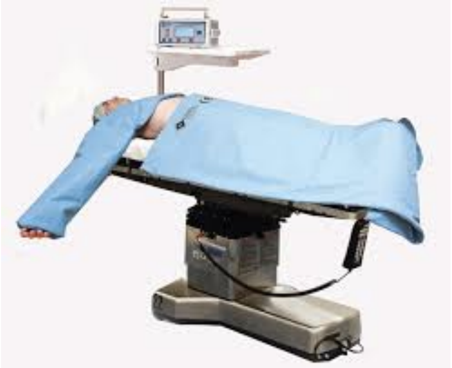

Direct contact resistive fabric warming (RFW) works like a low voltage electric blanket. A series of plastic coated, individually computer-controlled heating pads are used to warm the skin by direct contact. The pads can be placed both under the patient and over the parts of the body away from the operating site.

Who is invited to take part in this study?

This study is for patients over the age of 60 years who have suffered a broken hip and for whom there is a plan for surgery to repair or replace the broken bone. Most patients will have surgery as soon as possible after being admitted to hospital.

What happens to those who participate?

Whenever possible, a participant will be asked to sign a consent form after reading this booklet and deciding that they wish to take part. However, this may not always be possible because of the urgent need for surgery. In this emergency setting, we will seek verbal advice from a consultee to act on behalf of the patient. The consultee could be a relative, friend or medical doctor but not the chief investigator or other principal investigators conducting this trial. In situations when a consultee has advised that a patient would likely have agreed to participate, they will be given this booklet after their surgery and allowed time to consider if they wish to continue with the trial. For all participants, three copies of a consent/consultee advice form will be made: one for the participant to keep, one for the hospital medical records and one for the study site file.

All patients will undergo surgery as normal but those in the trial will be randomly allocated to either FAW or RFW prior to surgery. Neither the participant, a consultee, or the medical team providing care will be able to choose the warming method used. Each participant has an equal chance of being allocated to either group. After surgery, participants will be involved in the trial for up to three months but, during this time, they will not need to attend any extra clinics or provide any additional blood samples beyond what is required for their routine care. Participants will be asked just after their surgery, at 1 month and at 3 months about their well-being. An on-line questionnaire can be completed if they prefer. If we cannot contact a participant directly at 1 month and 3 months, we will contact their GP instead. We may seek further details from a participant's medical records, or talk to them directly, if they are unwell or admitted to hospital for any reason. Throughout this study, all participants will be treated with the same standard of care given to patients who are not involved in the trial.

Is participation compulsory?

No. Taking part in this study is entirely voluntary. A participant can withdraw from the trial at any time without a reason. Ongoing medical care will not be affected in any way. Likewise, a consultee can withdraw their advice at any time if they change their mind.

What else needs to be considered?

Taking part in this study will not affect any other medical conditions or medication that needs to be taken. If, for some reason, a hip fracture patient does not undergo surgery, they will not be able to take part in this study.

Will General Practitioners/family doctors (GP) be informed?

Yes. A participant's GP will be notified that their patient is involved in this study. We may telephone GPs if we cannot contact participants for the follow-ups at 1 month and 3 months.

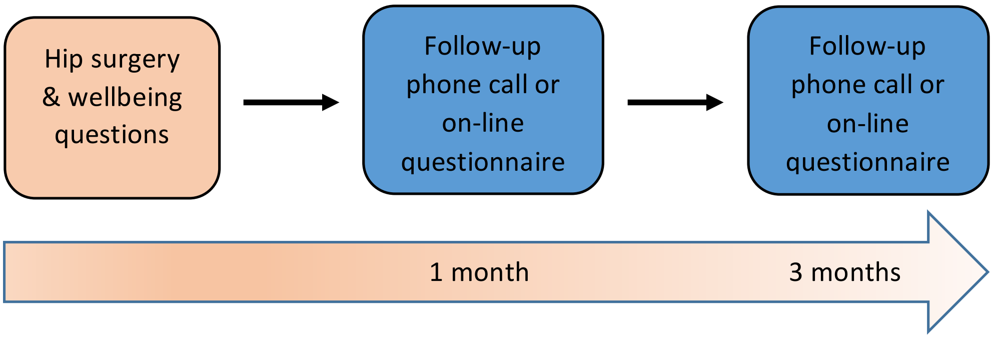

Are there any possible disadvantages or risks from taking part?

No. There are no extra risks associated with taking part in this study. Participants will be kept warm during surgery regardless of the group they are allocated to. The questions that will be asked about their wellbeing are to see if a participant has developed a post-operative infection. All participants will still be assessed by their medical care team following their operation in the normal way.

Are there any benefits of taking part?

No. As we do not know whether one warming method is better than the other in reducing the risk of post-operative infection, there are no known benefits for participants in either group. However, both groups will be helping to inform NHS practice in reducing the risk of post-operative infection for future patients. Participants will not be paid for taking part in this trial.

Will taking part in this study be kept confidential?

Yes. All study information will be stored securely on NHS or University computers in accordance with relevant data protection laws. No one outside of a participant's medical care team, the research team and the regulatory agencies governing the research will know who is taking part in this study. Anonymised data will be held for five years after the end of the study. Personal data, such as contact details and signed consent forms, will be stored in the study file in accordance with the Data Protection Act. These will be kept in a secure area within the hospital for up to six months after the study ends, after which point they will be securely destroyed.

What happens to those who change their minds and don't want to carry on with this study?

Participants or their consultees can withdraw from the study at any time and without giving a reason. The patient's medical care will not be affected in any way and treatment will continue as normal. We will use the data we have collected up to the point of withdrawal from the study unless a participant or their consultee tell us not to do so.

What will happen to the results of this study?

This pilot study will be used to support the development of a larger trial in many hospitals. All data will be incorporated into the larger, multi-centre trial which will be published in medical journals and presented at conferences. Participants will not be identified in any publications without their specific consent.

What if there is a problem?

Brighton and Sussex University Hospitals NHS Trust, as Sponsor, has insurance in place in the unlikely event that anyone suffers any harm as a direct consequence of this study. Anyone who wishes to complain about any aspect of the way in which they have been approached or treated during the course of this study, should contact the Principal Investigator *[Site to add details]* or *[Site to add details]*. NHS indemnity operates irrespective of the warming method used. Anyone who has cause for concern regarding the care they receive as an NHS patient can contact the Patient Advisory Liaison Service (PALS) *[Site to add details]*. PALS is a confidential NHS service that provides support for complaints and queries. PALS cannot provide specific information about this research study.

Who is organising and funding this study?

This study is being funded by the Healthcare Infection Society and by the company 3M™ (Patient Warming Solutions). It is being organised by the Brighton & Sussex Clinical Trials Unit. None of the medical care teams involved in this study will be paid specifically for conducting this trial.

Who has reviewed this study?

This study has been reviewed and approved by the West Midlands - Coventry & Warwickshire Research Ethics Committee and the Health Research Authority (HRA). The trial has been designed with input from cross-specialty hospital consultants, patient representatives, a statistician and a clinical trials unit.

The following are links to general information about taking part in research:

www.crn.nihr.ac.uk/can-help/patients-carers-public/how-to-take-part-in-a-study/
www.nhs.uk/Conditions/Clinical-trials/Pages/Introduction.aspx

If you would like further information about this study, please contact:

Local Research Nurse

[Insert local contact details]

Study Coordinator

Dr Michelle Kümin
University of Oxford
michelle.kumin@eng.ox.ac.uk
07527617768

Thank you for reading this information sheet

Reducing Implant Infection in Orthopaedics

Chief Investigator:
Dr Matthew Scarborough
Microbiology, Level 7, John Radcliffe Hospital,
Headington, Oxford, OX3 9DU
01865 741166 Matthew.Scarborough@ouh.nhs.uk

Principal Investigator:
[site to add details]

PARTICIPANT CONSENT & CONSULTEE ADVICE FORM

Study Number ☐☐☐☐☐☐☐☐☐ | *Recruitment Site*

Participant's First Name | *Participant's Surname*

PART ONE

Consultee's Name & Relationship to Participant

Date verbal advice received ☐☐ / ☐☐ / ☐☐

PART TWO

Please initial each box

I confirm that I have read/been read the Participant Information Sheet (Version ______, dated ____/___/___) for the above study. I have had the opportunity to ask questions which have been answered satisfactorily.	☐
I understand that participation in this study is voluntary and that consent for continuing involvement in the study can be withdrawn at any time without giving any reason and without affecting ongoing medical care.	☐
I understand that participant's medical records may be reviewed by the researchers and other individuals responsible for oversight, governance and regulation of this study.	☐
I understand that personal identifying information can be collected, stored and used by the research team on the understanding that all such information will be treated strictly confidentially.	☐
I understand that participant's GP's will be informed of involvement in this study.	☐

I agree to participate in this study

	Date
Participant's Signature	☐☐ / ☐☐ / ☐☐
Name and signature of health professional receiving written consent	☐☐ / ☐☐ / ☐☐

I agree to my relative/friend/patient's continuing participation

	Date
Consultee's Signature	☐☐ / ☐☐ / ☐☐
Name and signature of health professional receiving written advice	☐☐ / ☐☐ / ☐☐
