# Supplementary material for: Reducing Implant Infection in Orthopaedics (RIIiO): a pilot study for a randomised controlled trial comparing the influence of forced air versus resistive fabric warming technologies on postoperative infection rates following orthopaedic implant surgery in adults
Source: Trials. 2018 Nov 19;19:640. doi: 10.1186/s13063-018-3011-y (PMC6245696; doi:10.1186/s13063-018-3011-y)

RIIiO Pilot Study PROTOCOL

Study Title: Pilot Study for a randomised trial comparing the influence of forced air versus resistive fabric warming technologies on post-operative infection rates following orthopaedic implant surgery in adults.

Short title: Reducing Implant Infection in Orthopaedics (RIIiO) Pilot Study

Ethics Ref: 16/WM/0451

HRA Approval Ref: 197521

Date and Version No: Version 4.0 (16th January 2018)

Chief Investigator:

Dr Matthew Scarborough MRCP, FRCPath, PhD
Consultant in Clinical Infection, Oxford University Hospitals Foundation NHS Trust
Microbiology, Level 7, John Radcliffe Hospital, Headington, Oxford, OX3 9DU
Mobile: 07872436461
E-mail: Matthew.Scarborough@ouh.nhs.uk

Principal Investigators:

Dr Jillian Hewitt-Gray
Consultant Anaesthetist, Horton General Hospital, Oxford University Hospitals Foundation NHS Trust

Mr Mike Reed
Honorary Senior Clinical Lecturer, University of Newcastle and Consultant Trauma and Orthopaedic Surgeon, Northumbria Healthcare NHS Trust

Dr C Mark Harper
Consultant Anaesthetist, Royal Sussex County Hospital, Brighton

Mr Oliver Pearce
Consultant Orthopaedic Surgeon, Milton Keynes University Hospitals NHS Foundation Trust

Mr Mark Dunbar
Consultant Orthopaedic Surgeon, Heart of England NHS Foundation Trust

Dr David Partridge
Consultant microbiologist, Sheffield Teaching Hospitals NHS Foundation Trust

Mr Andrew Smith
East Kent Hospitals University NHS Foundation Trust

**Co-Investigator**	**Dr Stephen Bremner**<br>**Trial Statistician, Brighton and Sussex Medical School**
**Co-Applicants**	**Prof Matthew Costa**<br>Professor Orthopaedic Trauma, University of Oxford and Honorary Consultant Trauma Surgeon, John Radcliffe Hospital, Oxford University Hospitals NHS Trust<br><br>**Dr Michelle Kümin**<br>Post-doctoral Scientist, Oxford University
**Sponsor:**	Brighton and Sussex University Hospitals NHS Trust
**Funders:**	Healthcare Infection Society (HIS), the company 3M™ (Patient Warming Solutions) and the Nuffield Benefaction for Medicine and the Wellcome Institutional Strategic Support Fund (ISSF)
**Clinical Trials Unit:**	Brighton and Sussex Clinical Trials Unit<br>Room 204 Bevendean House<br>University of Brighton<br>Falmer, BN1 9PH<br>Tel: 01273 641444

Investigator Agreements:

"We have read this protocol and agree to abide by all provisions set forth therein. We agree to comply with the International Conference on Harmonisation Tripartite Guideline on Good Clinical Practice".

Dr Matthew Scarborough

____________________	________________________	_____________
**Chief Investigator**	**Chief Investigator Signature**	**Date**

Dr Jillian Hewitt-Gray

____________________	________________________	_____________
**Principal Investigator**	**Principal Investigator Signature**	**Date**

Mr Mike Reed

____________________	________________________	_____________
**Principal Investigator**	**Principal Investigator Signature**	**Date**

Dr C Mark Harper

____________________	________________________	_____________
**Principal Investigator**	**Principal Investigator Signature**	**Date**

Mr Oliver Pearce

____________________	______________________	____________
**Principal Investigator**	**Principal Investigator Signature**	**Date**

Mr Mark Dunbar

____________________	______________________	____________
**Principal Investigator**	**Principal Investigator Signature**	**Date**

Dr David Partridge

____________________	______________________	____________
**Principal Investigator**	**Principal Investigator Signature**	**Date**

Mr Andrew Smith

____________________	______________________	____________
**Principal Investigator**	**Principal Investigator Signature**	**Date**

Conflicts of interest:

This pilot study will be funded in part by a grant from 3M. The company has no input into the study design or management and will not be involved in recruitment to the trial or analysis of the findings.

Mr Mike Reed has received speaker fees from Heraeus and research funding from Heraeus, 3m, Zimmer and Convatec.

Dr C Mark Harper has been loaned equipment by various companies and paid honoraria by 3M and Molnlycke.

TABLE OF CONTENTS

1. SYNOPSIS 6
2. ABBREVIATIONS 7
3. BACKGROUND AND RATIONALE 8
4. OBJECTIVES AND OUTCOME MEASURES 9
5. TRIAL DESIGN 10
6. PARTICIPANT IDENTIFICATION 12
7. STUDY PROCEDURES 12
8. WARMING METHOD and TEMPERATURE MONITORING 17
9. SAFETY REPORTING 17
10. STATISTICS AND ANALYSIS 18
11. ECONOMIC EVALUATION 19
12. DATA MANAGEMENT 20
13. QUALITY ASSURANCE PROCEDURES 20
14. ETHICAL AND REGULATORY CONSIDERATIONS 20
15. FINANCE AND INSURANCE 22
16. PUBLICATION POLICY 22
17. REFERENCES 22
18. APPENDIX A: PARTICIPANT FOLLOW-UP TELEPHONE EQ-5D-5L QUESTIONNAIRE 24
19. APPENDIX B: EQ-5D-5L TELEPHONE QUESTIONNAIRE 25
20. APPENDIX C: PROXY EQ-5D-5L QUESTIONNAIRE 32

AMENDMENT HISTORY

Amend-ment No.	Protocol Version No.	Date issued	Author(s) of changes	Details of Changes made
AM01	2.0	24 Jan 2017	Matthew Scarborough, Justine Boles and Michelle Kümin	-Title: Change in title to confirm that trial is randomised.<br>-Change of PI at Oxford.<br>-Addition of co-applicant.<br>-Confirmation that 3M had no input into design, management, recruitment or analysis in the trial.<br>-Wording of the primary objective and endpoints updated throughout to match the synopsis.<br>5.0 and 7.5 : Addition of baseline 5Q-5D-5L measures.<br>7.4: Clarification that sealed envelope preparation may be delegated by the statistician.<br>7.6: Further baseline measurements.<br>9.1: SAE reporting instructions updated.<br>10.1: If formal interim analysis is undertaken this will be

				by an independent statistician.<br>10.3: Updates to analysis plan.<br>11: New health economic section.<br>14.5: Addition of NHS number to the eCRF.<br>17: Additional references (health economics).<br>18: Original questionnaire as a research tool.<br>19: 5Q-5D-5L telephone questionnaire.<br>20: Proxy 5Q-5D-5L questionnaire for patients lacking capacity.<br>Other minor administrative updates throughout.
AM02	3.0	5th July 2017	Michelle Kümin and Matthew Scarborough	- Addition of Co-Investigator/Trial Statistician<br>1. Updated study end date<br>7.1 Provision for up to six further centres<br>8. Clarity of IPH definition for binary analysis
AM03	4.0	16th January 2018	Michelle Kümin	- Addition of additional principal investigators<br>- Addition of another funder<br>- Change of contact details for chief investigator

1. SYNOPSIS

**Study Title**	Pilot Study for a randomised trial comparing the influence of forced air versus resistive fabric warming technologies on post-operative infection rates following orthopaedic implant surgery in adults.
**Short title**	Reducing Implant Infection in Orthopaedics (RIIiO) Pilot Study
**Acronym**	*RIIiO Pilot Study*
**Trial Design**	Parallel group, open label 1:1 randomised pilot study comparing post-operative infection rates following orthopaedic implant surgery using direct contact Resistive Fabric Warming (RFW) or Forced Air Warming (FAW)
**Trial Participants**	Adults of 60 years or over undergoing hemiarthroplasty following hip fracture
**Planned Sample Size**	There is no upper limit for the sample size. Progression rules for the definitive trial will include a projection of 100 participants per year at each pilot site. Participants will be recruited over a minimum period of 12 months at each site.
**Follow-Up Duration**	90 days from the date of surgery
**Planned Trial Period**	14th November 2016 to 31st December 2018
**Primary Objective**	The primary objective of the pilot study is to inform data management and recruitment strategies for a definitive trial comparing infection rates following orthopaedic surgery with two warming technologies.<br>The primary objective of the full trial: "Is there a significant difference in the incidence of post-operative infection after hemiarthroplasty using Forced Air Warming (FAW) as compared to Resistive Fabric Warming (RFW)?"
**Secondary Objectives**	The secondary objective of the pilot study is to explore the feasibility of collecting resource use and quality of life data, to inform the design of the health economics component of the proposed fully powered trial.<br><br>The secondary objective of the full trial: "Is there a significant cost-effectiveness advantage to the NHS of using either direct contact resistive fabric warming (RFW) or forced air warming (FAW)?"
**Primary Endpoints**	Numbers recruited and observed event rate for definitive deep surgical site infection (SSI) within 90 days of surgery.
**Secondary Endpoints**	Superficial SSI, inadvertent perioperative hypothermia (IPH), length of hospital stay, patient reported outcome measures for quality of life score (EQ-5D-5L), resource utilisation and serious adverse events (SAEs) including death.
**Investigational Medicinal Products**	None

2. ABBREVIATIONS

BSUH	Brighton and Sussex University Hospitals NHS Trust
CDC	Centers for Disease Control
CI	Chief Investigator
CRF	Case Report Form
CTU	Clinical Trials Unit
DSMB	Data and Safety Monitoring Board
FAW	Forced Air Warming
GCP	Good Clinical Practice
GP	General Practitioner
HRA	Health Research Authority
ICF	Informed Consent Form
ICH	International Conference on Harmonisation
ICMJE	International Committee of Medical Journal Editors
IPH	Inadvertent Perioperative Hypothermia
ITT	Intention to Treat
NHS	National Health Service
NICE	National Institute for Health and Care Excellence
NIHR	National Institute for Health Research
NITCAR	National Infection Trainee Collaborative for Audit and Research
PI	Principal Investigator
PHE	Public Health England
PIS	Participant/ Patient Information Sheet
RAFT	Research and Audit Federation for Trainees network in anaesthetics
R&D	NHS Trust R&D Department
REC	Research Ethics Committee
RFW	Resistive Fabric Warming
SAE	Serious Adverse Event
SAP	Statistical Analysis Plan
SOP	Standard Operating Procedure
SSI	Surgical Site Infection
TARN	Trauma Audit and Research Network
TSC	Trial Steering Committee
UCV	Laminar flow Ultra Clean Ventilation

UKCIRG	United Kingdom Clinical Infection Research Group
WHiTE	World Hip Trauma Evaluation

3. BACKGROUND AND RATIONALE

Around 250,000 lower limb orthopaedic implants are performed in the UK each year, including 160,000 knee or hip arthroplasties and 70,000 hip fracture repairs or hemiarthroplasties. These are amongst the most cost effective interventions for any medical condition treated under the NHS. However, post-operative infection complicates 1-3% of joint implants in general and between 2.3% (passive surveillance) and 7.3% (active surveillance) of hip fracture repairs in particular (Dale et al 2011, Ridgeway et al 2005). Around half of these are classified as deep infections which almost always require repeat surgery, a prolonged course of antibiotics and extensive rehabilitation. Furthermore, patients with implant associated infections have a significantly worse long term outcome and mortality as compared to patients following uncomplicated implant surgery. The consequences of surgical site infection (SSI) in this group of patients are therefore considerable. Despite the limited risk of SSI for an individual, orthopaedics and trauma surgery was identified as the most common source of SSI in inpatients during a point prevalence survey in England in 2011 (Health Protection Agency 2011). Given that arthroplasty rate more than doubled between 2000 – 2009 in the USA and Denmark (Lamagni T 2014), it seems inevitable that, in the absence of new initiatives, implant related SSI will be an increasingly important burden to patients and to the health economy. Assessing available interventions aimed at limiting the incidence of SSI is therefore an urgent priority.

In response to the global threat of emerging antimicrobial resistance, health care services are under increasing pressure to reduce antibiotic consumption. The most effective way to achieve this is to reduce infection rates. This is particularly true of bone and joint infections which commonly require a course of antibiotic therapy lasting up to six months (Osmon et al 2013). Reducing overall exposure to antibiotics will reduce the threat of emerging resistance and is likely to limit the risks of health care associated infections such as *Clostridium difficile*, methicillin resistant *Staphylococcus aureus* (MRSA) and carbapenemase producing Enterobacteriaceae (CPE). The considerable concern over the potential threat that emerging antibiotic resistance poses to the safety and efficacy of surgical procedures will sustain the search for widely generalizable mechanisms to limit the risk of infection and improve patient outcomes.

There are a number of ways in which the risk of surgical site infection may be reduced. In orthopaedic surgery, use of laminar flow ultra clean ventilation (UCV) became standard practice in the UK following a large randomised trial of 8055 patients (Lidwell et al 1982) in which post-operative infection rate was halved. However, because UCV depends upon high volume and high velocity filtered air, it exacerbates loss of body heat during surgery so that patients are rendered at risk of inadvertent perioperative hypothermia (IPH). As well as being associated with greater blood loss, delayed anaesthetic recovery and an excess length of hospital stay, IPH also predisposes the patient to post-operative infection. This was initially demonstrated in an individually randomised trial involving 200 patients undergoing colorectal surgery, and confirmed in 'clean' surgery (hernia repair, varicose vein surgery and breast surgery) in a randomised trial involving 420 patients; both trials showed a relative reduction in the incidence of surgical site infection of well over 50% for patients maintained at or above a temperature of 36°C (Kurz et al 1996; Melling et al 2001). These findings have been extrapolated such that perioperative patient warming is now recommended for any operation lasting >30 minutes. NICE has produced guidelines specifically relating to the prevention of IPH during surgery and has specified the need for research on mechanisms and devices to achieve this (NICE Clinical Guideline April 2008).

In current clinical practice, there are two principal technologies for keeping patients warm during surgery: (1) Forced Air Warming (FAW) and (2) direct contact Resistive Fabric Warming (RFW).

FAW relies upon electrically heated air being delivered through a disposable hollow duvet which is placed over the patient. Warmed air then exits through holes over the patient's skin and warms the patient by convection. FAW is the most widely used active warming technology in the UK. It is incorporated into national recommendations for the prevention and management of inadvertent perioperative hypothermia, supported by evidence for both its clinical effectiveness and cost effectiveness (NICE Clinical Guideline April 2008).

RFW is similar in principle to an electric blanket. It uses low voltage DC current passing through semi-conductive polymer fibre fabric which warms the patient by conduction. In 2011 NICE issued technology guidance relating to a specific RFW product (NICE medical technology guidance 7 August 2011). NICE found sufficient evidence to suggest that its effectiveness was similar to that of FAW in maintaining core temperature during surgery. There was no statement as to its clinical effectiveness in reducing the risk of SSI.

There is limited evidence to guide the choice of active warming technologies. Theoretical studies looking at air movement and particulates in sham orthopaedic surgery have suggested that FAW may result in interference with ultraclean ventilation systems commonly used in orthopaedic surgery (McGovern et al 2011; Legg et al 2012) but this has not been proven to influence clinical outcome. Two relevant clinical studies have investigated perioperative warming specifically in relation to orthopaedic post-operative infection rates. The first was a non-randomised study involving 30 patients; no events were observed within the first six post-operative months in either the FAW group (20 patients) or the group which had no active intraoperative warming (10 patients) (Moretti et al 2009). The second study compared infection rates before and after a change from FAW to RFW in a single NHS Trust. The study collected data relating to 1,437 consecutive elective hip or knee replacements over a three-year period. The results suggested a significant reduction in deep post-operative infections rates (from 3.1% to 0.8% p=0.02) when FAW was replaced by RFW (McGovern et al., 2011). However, the outcome data are subject to potential confounding by other factors which may have changed during the study period. The findings, therefore, do not constitute sufficiently robust evidence upon which to base a change in practice.

Summary Justification for a Pilot Study and Progression Plan

We postulate that the risk of post-operative orthopaedic implant infection may be influenced by the choice of intraoperative warming technology. We plan to investigate this through a multi-centre superiority trial comparing FAW and RFW in adults undergoing hemiarthroplasty following hip fracture. Health economic evaluation will form the secondary aim of the study.

The biggest barrier to a successful funding application for this trial is the number of participants required. Hemiarthroplasty carries a risk of deep SSI of around 2.5%. To provide 90% power to demonstrate an absolute risk reduction of 1%, using a 5% significance level, a trial will need to recruit approximately 8630 participants over a 3-year period. To ensure a robust application, we will conduct a pilot study across a limited number of NHS sites to demonstrate that the recruitment strategy, randomisation process and follow-up assessments are appropriate and effective. Provided that no major strategic changes are required, we anticipate that refinements can be adopted and the study rolled out to additional centres without a prolonged break in recruitment at pilot centres. Pilot data will subsequently be transferred to the full trial and will contribute to the final analysis.

The United Kingdom Clinical Infection Research Group (UKCIRG) is a research collaboration consisting of 43 NHS trusts in the UK. Its current interventional trials include The OVIVA Study (Oral vs. Intravenous Antibiotics in bone and joint infection) which recruits at 27 centres, and the ARREST Trial (Adjunctive Rifampicin to Reduce Early mortality in Staph aureus bacteraemia) which recruits at 30 centres. The WHiTE cohort is a collaboration of 16 NHS trusts recruiting patients under a single comprehensive treatment pathway, based upon the NICE Hip Fracture Guidelines, and provides core outcome measurements collected within the framework of the UK National Hip Fracture Database. Assuming that 30 of the centres from these networks are willing to participate, progression from pilot to a definitive trial will be considered if recruitment at each pilot centre exceeds an average of 2 participants per week or projects to over 100 per year after six months of activity. A second progression target designed to assess data management strategy will be defined by follow-up data for primary endpoints at Day 30 being available for >90% of participants six months after the start of the pilot study.

4. OBJECTIVES AND OUTCOME MEASURES

Primary Objective The primary objective of the RIIiO pilot study is to inform and assess the recruitment and data management strategies for a full trial.

Secondary Objectives The secondary objective of the pilot study is to confirm the methodology for health economic evaluation in the context of the full trial.

**Follow-Up Time Points**	Participants will be followed up at 30 days and 90 days following surgery.
**Primary Outcome Measure**	Recruitment rate and data completion (pilot study) and definitive deep surgical site infection (SSI) within 90 days of surgery (main trial).
**Secondary Outcome Measures**	Superficial SSI, documented IPH, length of hospital stay, patient reported outcome measures for quality of life score (EQ-5D-5L), resource utilisation and serious adverse events (SAEs) including death.

5. TRIAL DESIGN

The overall trial design, depicted in the flowchart below, is a parallel group, open label study randomising to RFW or FAW in permuted blocks in adults aged 60 years or over who are undergoing hemiarthroplasty following hip fracture. The trial has been designed with input from cross-specialty hospital consultants, patient representatives, a statistician and a clinical trials unit. This pilot study will recruit for a minimum of one year at each site, allowing for refinements to design, site selection and, if necessary, the data management strategy in preparation for a large multi-centre trial.

Participants will be expected to remain in the trial for approximately three months. They will not need to attend any research specific clinics or undergo any study specific clinical investigations. The primary endpoint is numbers recruited and observed event rate for definitive deep surgical site infection (SSI) within 90 days as defined by the Centre for Disease Control (CDC). All potential primary endpoints will be confirmed by a blinded endpoint committee. The secondary endpoints include serious adverse events including death, superficial SSI, IPH, length of hospital stay, patient reported outcome measures (EQ-5D-5L) and resource utilisation. Data for all assessments will be captured either from routine clinical care records or by telephone contact with participants or their GP practice at baseline, 30 (+/- 7) days and 90 (+/- 14) days. Patients will be flagged and mortality checks performed at each time point before the local study team makes contact for follow-up. Where a potential deep SSI is identified, a summary of the clinical care record, redacted for all personal identifiable information and any indication of randomisation arm, will be forwarded to the independent blinded endpoint committee.

RIIiO Pilot Study Flowchart

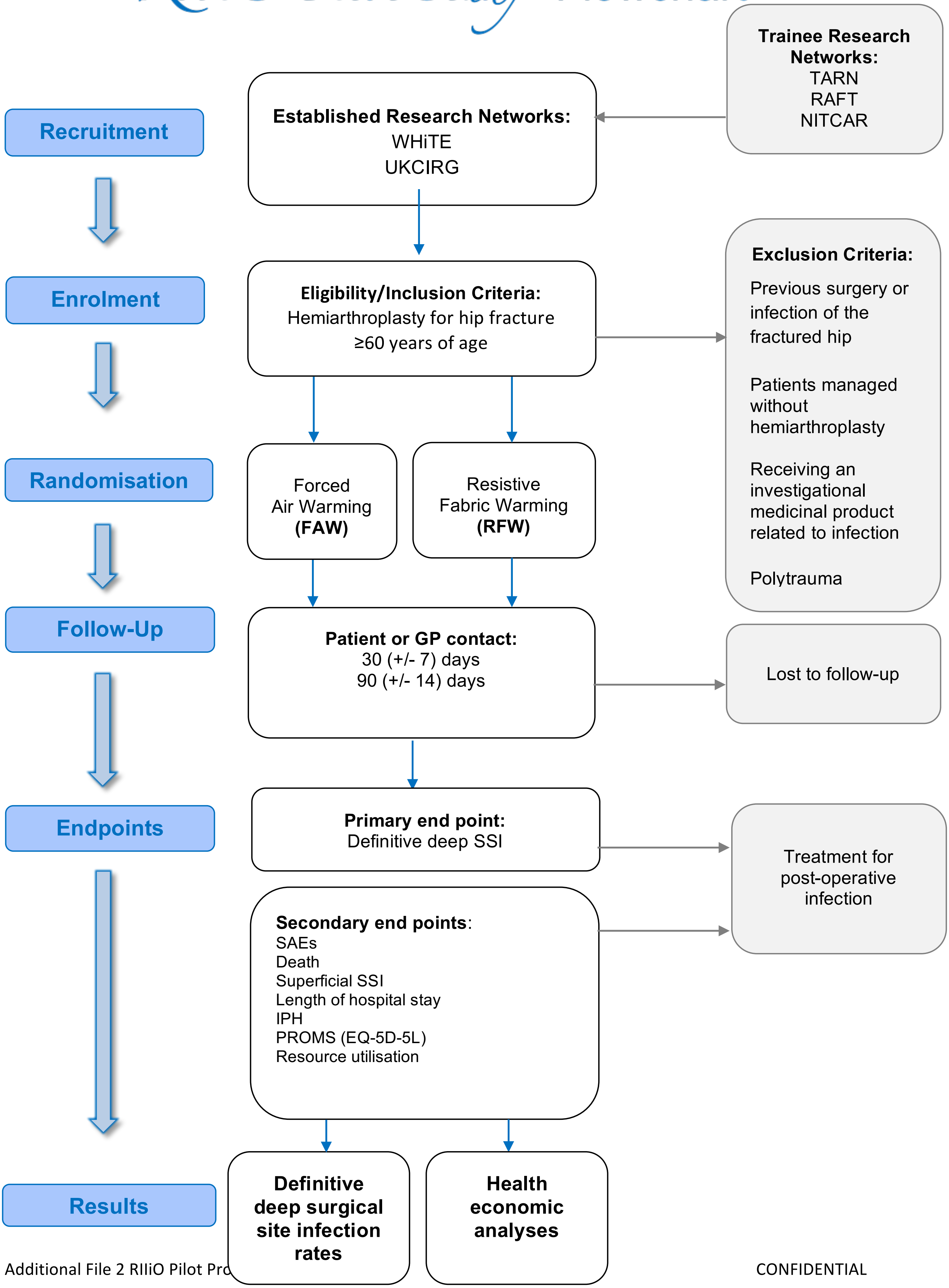

Endpoint Committee

The endpoint committee, which will remain blind to allocation, will comprise clinicians with expertise in the diagnosis and management of bone and joint infection. The committee will have a chair and two other members. If any endpoint committee member stands down during the course of the trial, they will be replaced by someone with similar background and qualifications.

Any post-randomisation re-admission, clinic attendance or return to theatre with signs and symptoms at the site of surgery (i.e. possible deep surgical site infections) will be considered a potential primary endpoint. A summary of the relevant medical record, redacted for personal identifiers and information relating to randomisation, will be forwarded to the blinded endpoint committee who will determine if an endpoint has been met. Determination of an endpoint will be by consensus following discussion or by a majority vote called by the chair if consensus cannot be reached.

Secondary endpoints, including SAEs and data for resource utilisation, will be determined directly by the local investigators.

6. PARTICIPANT IDENTIFICATION

6.1. Study Participants

This study will recruit trauma patients, aged 60 years or older, undergoing hemiarthroplasty following hip fracture.

6.2. Inclusion Criteria

The participant must meet ALL of the following criteria:

1) Provision of informed consent OR consultee declaration
2) Aged 60 years or over
3) Presenting with fracture of the hip
4) Scheduled to undergo hemiarthroplasty

6.3. Exclusion Criteria

The participant may not enter the study if ANY of the following apply:

1) Previous surgery or infection of the affected hip
2) Hip fractures related to polytrauma
3) Patients managed without hemiarthroplasty
4) Receiving an investigational medicinal product related to infection

7. STUDY PROCEDURES

7.1. Recruitment

The pilot study will recruit at three centres (Brighton, Oxford and Northumbria) in the first instance, each of which will aim to recruit a minimum of 100 participants in 12 months. Provision will be made to include up

to six further centres after six months. This will provide a mechanism to most accurately determine the total number of sites required for the definitive trial.

Research networks -

Recruitment to the definitive trial will take place through specialist centres drawn from the following networks:

1) The World Hip Trauma Evaluation (WHiTE) cohort, which is a comprehensive cohort study of patients with hip fracture treated at 16 specialist units in England. Since May 2015, WHiTE has recruited in excess of 300 patients per month. All patients are treated under a single comprehensive treatment pathway based upon the NICE Hip Fracture Guidelines and have core outcome measurements collected within the framework of the UK National Hip Fracture Database.
2) The United Kingdom Clinical Infection Research Group (UKCIRG) is a network of NHS infection specialists with specific expertise in the prevention, diagnosis and treatment of SSI. It recruits at 43 centres across the UK. UKCIRG studies currently include a 27-centre intervention trial looking at the management of orthopaedic infections (OVIVA), and a 30-centre intervention trial investigating adjunctive therapy in Staphylococcus aureus bacteraemia (ARREST).

Trainee networks -

Recruitment to both the pilot study and to the definitive trial will promote trainee network engagement in clinical research as part of improving research accessibility with NHS practice, and will provide a mechanism for optimising screening and recruitment at individual sites.

1) The Trauma Audit and Research Network (TARN)
2) The Research and Audit Federation for Trainees (RAFT) network in anaesthetics
3) The National Infection Trainee Collaborative for Audit and Research (NITCAR)

7.2. Screening and Eligibility Assessment

Potential participants will be identified during their routine care pathway from admission records, theatre lists and at daily trauma meetings at each recruiting site. Determination of eligibility for this study will be based on a review of the case notes and a clinical assessment in relation to the inclusion and exclusion criteria defined above. There are no other specific screening investigations and no additional laboratory or diagnostic tests will be required.

7.3. Informed Consent

Patients with a hip fracture are a clinical priority for urgent operative care and will usually undergo surgery on the next available operating list. Such patients have a high incidence of comorbidities, will inevitably have suffered trauma and are likely to either be in pain or to have received opiate analgesia. It is therefore understandable that patients find their initial treatment in hospital frightening, confusing or disorientating. In this situation, the focus necessarily lies on obtaining consent for surgery (where possible) and on informing the patient and next of kin about the immediate clinical care plan. It is often either inappropriate or not possible to ask potential participants to review trial documentation, weigh up the information and communicate an informed decision as to whether they would wish to participate.

Conducting research in an emergency setting is regulated by the Mental Capacity Act 2005. Given the number of factors influencing capacity or the ability to communicate an opinion, we will act in accordance with section 32, subsection 9b of the Mental Capacity Act. Those patients who are listed for surgery on the next available operating list will not be approached for consent prior to their surgery but we will approach an appropriate consultee. They will be provided with the study information and be given the opportunity to ask questions and discuss the study, after which their verbal agreement will be recorded. Where possible and appropriate, a personal consultee will be approached. Where necessary, a nominated consultee will be identified to advise the research team. The nominated consultee will be a clinician responsible for the

patient during their admission unless they are also a member of the research team, in which case an independent clinician will be asked to provide an opinion.

At the earliest appropriate opportunity after recovery from surgery, a member of the research team will approach randomised participants to provide them with study information and to seek written personal consent to continue in the study. They will be given as much time as they wish to discuss the study, to ask questions and talk to their family and carers. For those who have persisting lack of capacity, written agreement from a personal consultee will be sought. Participants (or their representatives) who do not wish to be contacted or who do not wish to complete questionnaires will be asked for consent to allow the research team to access and use routinely captured NHS data; these may include data recorded by their GP, the National Hip Fracture database and the Surgical Site Surveillance database. Alternatively, the participant (or their representative) can decline participation completely. The Information Sheet, consent form and consultee declaration state clearly that participants or consultees can withdraw their consent or advice at any time without giving a reason.

Signed consent forms will be stored in the investigator site file. A copy will be given to the participant (or their representative) and a copy will be placed in the participant's medical notes.

On occasion, patients may be able to provide consent before their operation, for example those whose surgery is delayed for clinical reasons. These patients will be approached prior to surgery for consent to participate in the study. If, despite delayed surgery, a patient lacks capacity the research team will approach a consultee.

Best efforts will be made to involve participants who, temporarily or permanently, lack capacity to make an informed decision. The research team will make a judgement as to the amount and complexity of the information that the patient is able to understand and retain on an individual basis. Appropriate information will be communicated to the participant and updated as their understanding changes. At all times the study team will act in accordance with the participant's best interests.

Any new information that arises during the study that may affect participants' willingness to take part will be reviewed by the TSC; if necessary this will be communicated to all participants and a revised consent form prepared.

Responsibility for documenting informed consent or agreement will lie with the investigator or persons designated by the investigator who conducted the consent discussion. The person who receives consent will be suitably qualified, experienced and authorised to do so by the Principal Investigator. Designated responsibility will be recorded in the site delegation log.

7.4. Randomisation and limitation of bias

Prior to surgery, participants will be randomised 1:1 in randomly permuted blocks through an established software package to either Forced Air Warming or direct contact Resistive Fabric Warming. No stratification factors will be employed. In case of software failure, randomisation envelopes will be prepared in advance under the supervision of a qualified statistician and held by the local study team for immediate use in the emergency setting.

This will be an open label trial. Although participants will not be directly informed of their randomised allocation, they may become aware of this either immediately before or upon recovery from anaesthesia. Similarly, it is not possible to blind the direct medical care team or the local study team as to which warming technology is utilised during surgery. Any consequent risk of bias will be limited by the use of a blinded end point committee for the assessment of relevant potential endpoints. For all potential deep SSIs, the end point committee will be provided with a summary of the participant's medical records relevant to the clinical episode, (including presentation, operative findings, laboratory results, radiology reports and treatment) redacted for personal identifiers and any information relating to their randomisation or intraoperative thermoregulation. At the time of randomisation, the participant's initials, NHS number and a computer-generated study number will be recorded on an enrolment log. Justification for use of NHS numbers comes from previous multicentre studies in which the randomisation software requires all

participants to have a primary identifier of the same alphanumeric structure and with no opportunity for duplicates.

7.5. Baseline and Perioperative Assessments

Data for baseline assessments will be captured from routine clinical care records. The local study team will record the following in the CRF:

1) Age
2) Gender
3) Weight/Height (to derive Body mass index)
4) American Society of Anaesthesiologists (ASA) physical status classification
5) Anatomical side affected
6) Date of admission
7) Date of surgery
8) Randomisation arm (FAW or RFW)
9) Adherence to randomised strategy
10) Duration of surgery
11) Use of UCV in theatre
12) Surgical procedure
13) Antimicrobial prophylaxis
14) Immuno-suppressants
15) Co-Morbidities
16) Continuous temperature monitoring (3M SpotOn Zero Flux Thermometry)
17) Baseline EQ-5D-5L measures

7.6. Subsequent Assessments

The participant will be contacted for follow-up at the following time points after surgery:

1) 30 (+/- 7) days
2) 90 (+/- 14) days

Follow-up assessments will be undertaken by a member of the local study team through a) medical records and b) direct contact, usually by telephone, with the participant or their consultee. The participant will not be asked to attend any research specific clinic visits and they will not be visited at home by the study team. If it is not possible to make contact with the participant, the local study team will gather follow-up data from the patient's GP and from routinely collected surveillance data. At each time point, we will ask questions about the participant's wellbeing, surgical scar and any treatment or concerns around possible infection at the operative site. Where necessary, further clinical review will be requested.

The study will use the participant follow-up telephone questionnaire in Appendix A as a research tool when gathering patient reported outcome measures for quality of life (PROMS) and any relevant concerns they have. Instructions to the participant will be dictated from the EQ-5D-5L telephone questionnaire in Appendix B. If a patient lacks capacity, the EQ-5D-5L Proxy version 1 will be used (Appendix C).

Deep SSI will be defined by the following criteria:

a. Infection arising within 90 days of the index surgery (where day 1 is the procedure date) AND
b. Involves deep tissues related to the incision (e.g. fascial and muscle layers, joint space or periprosthetic region)

AND

c. At least one of the following

    i. Purulent drainage from the deep incision or periprosthetic drain.

    ii. A deep incision that spontaneously dehisces, or is deliberately opened or aspirated or biopsied by a surgeon, physician or other designee and an organism is identified by a culture or non-culture based microbiologic testing method which is performed for purposes of clinical diagnosis or treatment (e.g., not Active Surveillance Culture/Testing) or culture or non-culture based microbiologic testing method is not performed

    iii. An abscess or other evidence of infection involving the deep incision or periprosthetic region that is detected on gross anatomical, histopathological exam or imaging test

Superficial SSI will be defined by the following criteria:

a. Infection arising within 30 days of the index surgery (where day 1 is the procedure date)
   AND
b. Involves only skin or subcutaneous tissue related to the incision
   AND
c. At least one of the following

    i. Purulent drainage from the superficial incision

    ii. Organisms identified from an aseptically-obtained specimen from the superficial incision or subcutaneous tissue by a culture or non-culture based microbiologic testing method which is performed for purposes of clinical diagnosis or treatment (e.g., not Active Surveillance Culture/Testing (ASC/AST).

    iii. Superficial incision that is deliberately opened by a surgeon, physician or other designee and culture or non-culture based testing is not performed
    AND
    the patient has at least one of the following signs or symptoms:
    pain or tenderness, localized swelling, erythema, heat

    iv. Diagnosis of a superficial incisional SSI by a surgeon or physician.

The above definitions are adapted from the CDC surgical site infection criteria published January 2016. If required for clarification or consensus, investigators and the EPC will be referred back to the source document.

Serious adverse events (SAEs), including death (i.e. all-cause mortality), will be recorded.

Discontinuation/Withdrawal of Participants from Study

Once a participant has been randomised, they will ordinarily be included in the intention to treat (ITT) analysis. However, the Principal Investigator at each site will withdraw any randomised participant from the study if they do not subsequently undergo surgery. Each participant or their consultee has the right to withdraw from the study at any time with no obligation to give a reason for withdrawing. The participant will be asked whether or not they will allow passive follow up using routinely available NHS data. All data collected up until the point of withdrawal of consent will be included in the final analysis unless requested otherwise by the participant.

7.7. Definition of End of Study

The end of the study is defined as the date of the last follow-up telephone call to the last participant recruited. Any planned formal analyses of the data will take place after this time point.

8. WARMING METHOD and TEMPERATURE MONITORING

The participants recruited to this study will all undergo hemiarthroplasty. During their surgery the patient will be kept warm as part of their standard care. The primary method used will be either Forced Air Warming (FAW) or direct contact Resistive Fabric Warming (RFW) depending on the randomisation arm to which they have been allocated. Both FAW and RFW are established and licensed for use in the UK and are equally effective at preventing inadvertent perioperative hypothermia (Negishi et al.2003). The warming devices will be used in accordance with national guidelines as defined in NICE CG65. Where necessary for optimal clinical care, additional warming methods can be employed at the discretion of the supervising clinician. Standard clinical care would normally include actively warming intravenous fluids and blood products in all patients.

Temperature will be monitored throughout the surgical procedure at all recruiting sites. All thermometers will be calibrated according to the standard protocol at each site. Where possible, site will use the 'SpotOn zfd' temperature monitoring system provided by the company 3M™ (Patient Warming Solutions).

As part of standard clinical care the patient's temperature will be measured and documented before induction of anaesthesia and every 30 minutes until the end of surgery. For the binary analysis, IPH will be defined as a temperature of <36ºC at the end of surgery.

In addition, we will use the SpotOn zfd to log a continuous readout to provide the following data:

a) IPH at any time during surgery *and* at the end of surgery/arrival in the recovery room
b) Area under the curve for total hypothermia time during surgery
c) Weighted hypothermia time (i.e. time spent at 35.5ºC is more significant than time spent at 35.9ºC)

9. SAFETY REPORTING

A serious adverse event is defined as any untoward medical occurrence that:

- results in death OR
- is life-threatening OR
- requires inpatient hospitalisation or prolongation of existing hospitalisation OR
- results in persistent or significant disability/incapacity

Other 'important medical events' may also be considered serious if they jeopardise the participant or require an intervention to prevent one of the above consequences.

NOTE: The term "life-threatening" in the definition of SAE refers to an event in which the participant was at risk of death at the time of the event; it does not refer to an event which hypothetically might have caused death if it were more severe.

9.1. Reporting Procedures for Serious Adverse Events

All SAEs will be recorded in the CRF. An SAE occurring to a participant will be reported to the sponsor, the Data and Safety Monitoring Board (DSMB) (see section 12) and the REC when, in the opinion of the Chief Investigator, the SAE is **unexpected** and **related** to the allocated intraoperative warming technology. Such SAEs will be reported using the sponsor's SAE report form and emailed to safety@bsuh.nhs.uk within 24 hrs of becoming aware of the event. If required, the SAE will be reported to the appropriate bodies within 15 days of the CI becoming aware of the event.

The following will be considered as expected SAE's:

1) Complications of hip fracture and hip fracture surgery (including anaesthesia)
2) Complications arising as a result of inadvertent perioperative hypothermia
3) Heat necrosis and pressure areas acquired during the clinical episode relevant to the study
4) Inter-current illnesses causally related to comorbid conditions that the investigator believes are likely given the patient's history, age and other factors

The investigators will use their judgement, such that SAEs technically meeting the definitions above but that seem unexpected in terms of severity, duration or other factors may be regarded as unexpected. All participants experiencing SAEs will be followed-up as per protocol until the end of the trial.

Documented endpoints, including superficial or deep SSI and IPH (but excluding death), will not be reported as SAEs.

10. STATISTICS AND ANALYSIS

10.1. Pilot study

The primary objective of the pilot study is to inform accrual expectations and data management strategies for a definitive trial. A formal interim analysis of efficacy will only be invoked if recruitment to the pilot study exceeds 1000 patients. This will be the responsibility of an independent statistician.

Outcome data (including SAEs) and compliance will be summarised according to the randomised intervention. Measures will be quantified and, where appropriate, a 95% confidence intervals will be presented. No imputation for missing data will be performed for the pilot study.

Estimates of recruitment rates, and the rates of definitive deep surgical site infection within 90 days of surgery, will be used to further inform any changes to design, data management and recruitment strategy for the main study.

The DSMB will review interim summaries for accrual, safety, conduct and, if appropriate, outcome. In the light of the data presented, and any additional information that they require, the DSMB may recommend amendment, suspension or closure of the study to the Trial Steering Committee (TSC).

10.2. The Number of Participants

The primary objective of the pilot study is to demonstrate that strategies for recruitment and data management are appropriate and robust. There is, therefore, no defined upper limit for the number of participants required. The study will recruit at three sites initially, each of which performs between 4 and 8 hemiarthroplasties per week. Participants will be recruited over a minimum period of 12 months at each site and the number extrapolated to determine recruitment strategy, including expected number of sites, for a full trial.

10.3. Analysis of Outcome Measures for the full trial

Pending recommendations arising from the pilot study, we assume that the analysis of outcome measures for the full trial will take the following form:

<u>Analysis of the primary endpoint:</u>
Based on the ITT population (i.e. participants analysed according to their allocated trial arm, regardless of their compliance with the protocol), the proportions of participants in either treatment arm experiencing a definitive deep surgical site infection within 90 days from surgery will be calculated.

We will determine the superiority of either of the two trial interventions by assessing if there is a statistically significant difference in the log-odds of infection between the randomised treatment arms using a logistic regression model with randomised treatment as a fixed effect; superiority of either intervention is defined by the treatment coefficient being statistically significant at the 5% level. Covariates considered *a priori* to be prognostic of outcome may be included to give an adjusted odds ratio. These would be agreed by consensus and written into the full statistical analysis plan and signed off prior to analysis of unblinded data.

Secondary analyses will include a per protocol analysis based on all participants who have received their randomised intervention. A survival analysis will be performed to assess potential differences in the time to diagnosis of definitive deep surgical site infections. Health economic evaluation will be conducted by a dedicated health economist based on capital, maintenance and treatment costs, as well as EQ-5D-5L data, and will include the costs of complications.

Other secondary endpoint analyses will include regression models to calculate estimates of treatment differences for the primary and secondary outcomes unadjusted (primary analysis) and adjusted for age and co-morbidities (secondary analyses). These secondary analyses will focus on the consistency of point estimates and 95% confidence intervals rather than formal statistical significance testing.

Pre-defined subgroup analyses will use interaction tests to explore the consistency of treatment effects by trial site.

Patients who are randomised but, for whatever reason, do not proceed to surgical management will be excluded from all analyses. For patients who withdraw their consent, no further data will be collected from follow-up questionnaires, but data collected up to the point of withdrawal and routine data from medical records will be included unless the participant denies access to their medical records.

11. ECONOMIC EVALUATION

Good practice recommendations for cost-effectiveness analyses (Ramsey et al, 2015) suggest concentrating on the measurement of large cost drivers, with less focus on resources that are not expected to differ between different treatments. Estimation of cost-effectiveness is therefore an iterative process and, by including a health economic component, it is possible to consider how the methods might be refined in any future more definitive study.

Methods

In order to estimate costs, we will measure theatre time, length of stay and hospital re-admissions. Unit costs (e.g. Curtis et al, 2015, Dept of Health, 2014-2015) will subsequently be assigned to items of resource use. However, in order to estimate the unit costs associated with both the RAW and FAW intervention, each site will be asked to complete a short questionnaire which would request information such as the purchase price of intervention specific equipment and whether items are disposed of after each patient or re-used.

For benefits, the EQ-5D-5L (Herdman et al, 2011) will be used to measure quality of life, enabling QALY (Quality Adjusted Life Year) scores to be calculated. This will be requested at baseline, day 30 and day 90. The self-complete version of the EQ-5D-5L will be used for those who give consent, consultees will be asked to complete the proxy version of the EQ-5D-5L.

Analysis

The main purpose of the analysis is to inform the decision regarding how and what cost and effect data would be collected within the proposed fully powered trial. Thus, we will estimate completion rates and seek to identify big cost drivers, in order to inform this decision.

12. DATA MANAGEMENT

12.1. Access to Data

Direct access will be granted to authorised representatives from the Sponsor, appropriate regulatory authorities and host institution for monitoring and/or audit of the study to ensure compliance with regulations.

12.2. Data Recording and Record Keeping

Electronic CRFs will be completed by a member of the local study team using data collected from either review of routine medical records or direct contact with the participant, their consultee or their GP. Access to the electronic database management system (MACRO) will be restricted by password to authorised users and software protection applied in accordance with national data protection standards. Hard copy screening and enrolment logs will be kept within the local Trust under appropriate conditions (e.g. locked filing cabinet or locked office) such that access is restricted to the local study team and authorised personnel only. All trial data will be archived for 5 years following completion of the study.

13. QUALITY ASSURANCE PROCEDURES

The study will be conducted in accordance with the current approved protocol, International Conference on Harmonisation (ICH), Good Clinical Practice (GCP), relevant regulations and standard operating procedures. The trial sponsor will have full and free access to all aspects of the trial to allow monitoring of compliance with regulatory arrangements. A trial steering committee (TSC) will be formed that will comprise at least an independent chair person, an independent deputy chair, two independent public/patient group representatives, the Chief Investigator, Principal Investigators and the trial co-ordinator. The TSC will meet either in person or by teleconference at the beginning of the pilot study, after eight months of recruitment and towards the end of the pilot to *(i)* review protocol amendments and/or deviations, *(ii)* make recommendations regarding the conduct of the trial, *(iii)* review recruitment and follow-up rates, and *(iv)* assess the progression plan to a full trial based on extrapolation of data acquired in the pilot study.

A data and safety monitoring board (DSMB), comprising at least three independent and suitably qualified persons, will meet (either in person or by teleconference) to discuss the study design and SOPs shortly before the start of the study. Investigators will participate in this meeting. The DSMB will also meet prior to each TSC meeting and at any other time they deem necessary to evaluate patient safety and frequency of endpoints in an un-blinded analysis. Investigators will not be present. The DSMB may make recommendations to the TSC at any time during the pilot study and before investigators proceed with the multicentre trial.

If more than 1000 participants are recruited to the pilot study, a formal analysis of efficacy will be undertaken for presentation to the DSMB. It is expected that they would only recommend suspending the study if there was a very significantly worse outcome in one arm (e.g. using the Haybittle-Peto stopping boundary) or concerns around patient safety in relation to the trial.

Both intraoperative warming technologies are CE marked and will be used within the marketing authorisations. Therefore, the study does not fall within the remit of the Medicines for Healthcare products Regulatory Agency.

Monitoring of the study will be in accordance with a risk assessment and monitoring plan as implemented by the CTU.

14. ETHICAL AND REGULATORY CONSIDERATIONS

14.1. Declaration of Helsinki

The Chief Investigator will ensure that this study is conducted in accordance with the principles of the Declaration of Helsinki.

14.2. ICH Guidelines for Good Clinical Practice

The Chief Investigator will ensure that this study is conducted in accordance with relevant regulations and Good Clinical Practice.

14.3. Approvals

The protocol, informed consent form, participant information sheet and any other patient or consultee documents will be approved by the Sponsor before being submitted to an appropriate Research Ethics Committee (REC) for written approval. No study procedures will take place before the REC has given a favourable opinion. The Chief Investigator will obtain written approval from the Sponsor and the REC for any substantial amendment to the original-approved documents before any changes are implemented.

14.4. Reporting

Throughout the study, the Chief Investigator will submit an Annual Progress Report to the REC and the Sponsor once a year, or more frequently on request. In addition, an End of Study Notification and Final Report will also be submitted to the REC and Sponsor within the required timelines. Any additional reports required will be submitted upon request (e.g. Trust R&D, funder).

14.5. Participant Confidentiality

The study team will ensure that each participant's participation is maintained confidential at all times. All documents and databases will be stored securely and will be accessible to study staff and authorised personnel only. The study will comply with the Data Protection Act, which requires data to be anonymised as soon as is practical. The participants will be identified by initials, NHS number and study number only on the electronic CRF. Encrypted administrative databases, maintained separately from the electronic CRFs, will contain each participant's contact details for the purposes of follow-up at each recruiting site. Access to the administrative databases will be restricted to the study teams. The electronic CRFs will be managed by the CTU. All databases will be archived for 5 years following completion of the study.
Inclusion of the NHS number reflects a requirement for the primary identifier to take a single alphanumeric format on the electronic database for randomisation, prevention of duplication and to allow for mortality checks prior to attempting to contact participants for follow up data. In the event that a participant has passed away, follow up data will be obtained through the GP by the local study team.

14.6. Expenses and Benefits

There will be no expenses or other payments to participants in this study. No visits additional to normal care are required so no expenses will be incurred. There are no know benefits for patients in either group but both groups will help to inform NHS practice in relation the risk of post-operative infection.

14.7. Other Ethical Considerations

The interventions (either FAW or RFW) in this trial are minimally invasive. Both technologies are licenced, equally effective at preventing inadvertent perioperative hypothermia and are in routine use in the NHS. There is no evidence to suggest that either method should be used preferentially for any individual or group of patients. Currently, the choice of warming method is governed by local availability or personal preference of the anaesthetic or surgical team involved. Outside the context of a trial, patients are generally unaware that active warming during surgery forms part of their care. As a consequence, and in

the absence of any trial specific clinic visits or clinical investigations, the consenting process is weighted on follow-up data (including access to healthcare records) and questionnaires.

Due to the inevitable history of trauma, high incidence of comorbidities and urgent nature of surgery, we believe it inappropriate routinely ask potential participants to review, weigh up and retain information relating to the trial preoperatively. We are likely therefore to seek consultee agreement in a significant number of participants. We feel this is justified on grounds that participants' optimal medical and surgical management, including psychological care, should not be delayed or jeopardised in relation to the trial. Precedent for this model of consent specifically in this patient group comes from the WHiTE cohort and associated randomised controlled trials (Griffin et al., 2013; Sims et al., 2016).

Of the three initial pilot sites, one currently uses FAW exclusively, one uses RFW exclusively and the third uses a combination of FAW and RFW according to preference of the anaesthetic and surgical teams. The investigators from all three sites have expressed equipoise in relation to the choice of warming technology and their associated efficacy and clinical outcomes.

15. FINANCE AND INSURANCE

15.1. Funding

This study is funded by the Healthcare Infection Society and by the company 3M^{TM} (Patient Warming Solutions).

15.2. Insurance

NHS Indemnity will apply.

16. PUBLICATION POLICY

The Chief Investigator, Principal Investigators and Sponsor will be involved in reviewing drafts of any manuscripts, abstracts, press releases or other publications arising from the study. Authors will acknowledge that the study was funded by the Healthcare Infection Society and the company 3M^{TM} (Patient Warming Solutions). Authorship will be determined in accordance with the International Committee of Medical Journal Editors (ICMJE) guidelines and other contributors will be acknowledged. All publications will be in open access form. The findings of the study, and any trial publications, will not be provided to participants but a summary of the findings in lay terms will be provided upon request. The sponsor will be kept informed of publications arising from this work.

17. REFERENCES

Brandt S, Oguz R, Hüttner H, Waglechner G, Chiari A, Greif R, Kurz A, Kimberger O. Resistive-polymer versus forced-air warming: comparable efficacy in orthopedic patients. Anesth Analg. 2010 Mar 1;110(3):834-8

Griffin XL, McArthur J, Achten J, Parsons N, Costa ML. The Warwick Hip Trauma Evaluation One – an abridged protocol for the WHiTE One Study: An embedded randomised trial comparing the x-bolt with sliding hip screw fixation in extracapsular hip fractures. Bone Joint Res 2013;2:206–9

Håvard Dale, Inge Skråmm, Hege L Løwer, Hanne M Eriksen, Birgitte Espehaug, Ove Furnes, Finn Egil Skjeldestad, Leif I Havelin & Lars B Engesæter Infection after primary hip arthroplasty. Acta Orthopaedica. 2011 82:6: 646-654

Kimberger O, Held C, Stadelmann K, Mayer N, Hunkeler C, Sessler DI, Kurz A. Resistive Polymer Versus Forced-Air Warming: Comparable Heat Transfer and Core Rewarming Rates in Volunteers. Anesth Analg. 2008 Nov 107(5):1621-1626

Klouche S, Sariali E, Mamoudy P. Total hip arthroplasty revision due to infection: a cost analysis approach. 2010 Orthop Traumatol Surg Res 96:124–32.

Kurz A, Sessler DI, Lenhardt R. Perioperative normothermia to reduce the incidence of surgical-wound infection and shorten hospitalization. N Engl J Med. 1996 May 9;334(19):1209-15

Lamagni T Epidemiology and burden of prosthetic joint infections J Antimicrob Chemother 2014; 69 Suppl 1: i5–i10

Legg AJ, Cannon T, Hamer AJ. Do forced air patient-warming devices disrupt unidirectional downward airflow? J Bone Joint Surg Br. 2012 Feb 94(2):254-6

Lidwell OM, Lowbury EJL, Whyte W, Blowers R, Stanley SJ, Lowe D. Effect of ultraclean air in operating rooms on deep sepsis in the joint after total hip or knee replacement: A randomised study. British Medical Journal. 1982 Vol.285(6334), pp.10-14

McGovern PD, Albrecht M, Belani KG, Nachtsheim C, Partington PF, Carluke I, Reed MR. Forced-air warming and ultra-clean ventilation do not mix: an investigation of theatre ventilation, patient warming and joint replacement infection in orthopaedics. J Bone Joint Surg Br. 2011 Nov 93(11):1537-44

Melling AC, Ali B, Scott EM, Leaper DJ. Effects of preoperative warming on the incidence of wound infection after clean surgery: a randomised controlled trial. Lancet. 2001 Sep 15;358(9285):876-80

Morettia B, Laroccab AMV, Napolib C, Martinellib D, Paolilloa L, Cassanoa M, Notarnicolaa A, Morettia L, Pescea V. Active warming systems to maintain perioperative normothermia in hip replacement surgery: a therapeutic aid or a vector of infection? J Hospt Infect. 2009 73;58-63

Negishi C, Hasegawa K, Mukai S, Nakagawa F, Ozaki M, Sessler DI. Resistive-heating and forced-air warming are comparably effective. Anesthesia and analgesia. 2003 June Vol.96(6), pp.1683-7

National Institute for Health and Care Excellence. Hypothermia: prevention and management in adults having surgery. April 2008. https://www.nice.org.uk/guidance/cg65/ (last accessed 28.6.16)

National Institute for Health and Care Excellence. Inditherm patient warming mattress for the prevention of inadvertent hypothermia August 2011. https://www.nice.org.uk/guidance/mtg7 (last accessed 28.06.16)

Osmon DR, Berbari EF, Berendt AR *et al*. Diagnosis and management of prosthetic joint infection: clinical practice guidelines by the Infectious Diseases Society of America. *Clin Infect Dis* 2013; **56**: e1–25

Ridgeway S, Wilson J, Charlet A, Kafatos, G, Pearson A, Coello R. Infection of the surgical site after arthroplasty of the hip. *J Bone Joint Surg* 2005 87-B:844-50

Sims AL, Parsons N, Achten J, Griffin XL, Costa ML, Reed MR. The World Hip Trauma Evaluation Study 3: Hemiarthroplasty Evaluation by Multicentre Investigation - WHITE 3: HEMI - An Abridged Protocol. Bone Joint Res. 2016 Jan;5(1):18-25. doi: 10.1302/2046-3758.51.2000473

Ramsey SD, Willke RJ, Glick H, et al. Cost-effectiveness analysis alongside clinical trials II—An ISPOR Good Research Practices Task Force Report. Value in Health 2015;18;161-72.

Curtis L, Burns A. Unit Costs of Health and Social Care 2015. University of Kent: 2015.

Department of Health. National Schedule of Reference Costs 2014-15. https://www.gov.uk/government/publications/nhs-reference-costs-2014-to-2015, 2015.

Herdman M, Gudex C, Lloyd A, et al. Development and preliminary testing of the new five-level version of EQ-5D (EQ-5D-5L). Qual Life Res 2011;20;1727-36.

18. APPENDIX A: PARTICIPANT FOLLOW-UP TELEPHONE EQ-5D-5L QUESTIONNAIRE

[Site to insert details]

RIIiO Pilot Study

Participant Follow-Up Telephone EQ-5D-5L Questionnaire

Study Number: ☐☐☐☐☐☐☐☐

Date of Completion: D D M M Y Y

Follow-up Time Point:
(Tick ONE)

30 (+/- 7) days ☐

90 (+/- 14) days ☐

SECTION 1

All of the questions in this section relate to the participant's health on the day that the questionnaire is completed

Mobility

- I have no problems in walking about ☐
- I have slight problems in walking about ☐
- I have moderate problems walking about ☐
- I have severe problems walking about ☐
- I am unable to walk about ☐

Pain/Discomfort

- I have no pain or discomfort ☐
- I have slight pain or discomfort ☐
- I have moderate pain or discomfort ☐
- I have severe pain or discomfort ☐
- I have extreme pain or discomfort ☐

Anxiety/Depression

- I am not anxious or depressed ☐
- I am slightly anxious or depressed ☐
- I am moderately anxious or depressed ☐
- I am severely anxious or depressed ☐
- I am extremely anxious or depressed ☐

Self-Care

- I have no problems with caring for myself ☐
- I have slight problems washing or dressing myself ☐
- I have moderate problems washing or dressing myself ☐
- I have severe problems washing or dressing myself ☐
- I am unable to wash or dress myself ☐

RIIiO Pilot Study Participant Follow-Up EQ-5D-5L Questionnaire v2.0 (20th January 2017)

[Site to insert details]

RIIiO Pilot Study

Usual Activities *(e.g. work, study, housework, family or leisure activities)*

- I have no problems with performing my usual activities ☐
- I have slight problems with performing my usual activities ☐
- I have moderate problems with performing my usual activities ☐
- I have severe problems with performing my usual activities ☐
- I am unable to perform my usual activities ☐

Health State

*Well-being score **TODAY** on a scale of 0-100, where 100 is the best possible health state and zero is the worst possible health state* ☐

SECTION 2

Read out the questions below. Record the participant's answers and any further comments they make

Surgical Wound

Have you had any concerns about the wound from your hip operation since you were last contacted by the RIIiO Study Team? Yes ☐ No ☐

Participant's Comments:

____

____

____

____

____

[Site to insert details]

RIIiO Pilot Study

Further Surgery

Have you had any further surgery on your affected hip since you were last contacted by the RIIiO Study Team?

Yes ☐ No ☐

If yes, what was the date(s) of the surgery? ....

Details of the operation:

____

____

____

____

____

Participant's Comments:

____

____

____

____

____

Form Completed by:

Print Name	Signature

[Site to insert details] *RIIiO Pilot Study*

Prescription(s)

Have you taken antibiotics for your wound since you were last contacted by the *RIIiO* Study Team? **Yes** ☐ **No** ☐

If yes, what was the date(s) of the prescription(s)? ....

Participant's Comments:

____

____

____

____

____

Readmission(s)

Have you been readmitted to any hospital since you were last contacted by the *RIIiO* Study Team? **Yes** ☐ **No** ☐

If yes, what was the date(s) of admission(s)? ....

Participant's Comments:

____

____

____

____

____

19. APPENDIX B: EQ-5D-5L TELEPHONE QUESTIONNAIRE

Health Questionnaire

English version for the UK

SCRIPT FOR TELEPHONE INTERVIEW

GENERAL INTRODUCTION

It is suggested that the telephone interviewer follows the script of the EQ-5D. Although allowance should be made for the interviewer's particular style of speaking, the wording of the questionnaire instructions should be followed as closely as possible. In the case of the EQ-5D descriptive system on pages 2 and 3, the precise wording must be followed.

It is recommended that the interviewer has a copy of the EQ-5D in front of him or her as it is administered over the telephone. This enables the respondent's answers to be entered directly on the EQ-5D by the interviewer on behalf of the respondent (i.e. the appropriate boxes on pages 2 and 3 are marked and the scale on page 4 is marked at the point indicating the respondent's 'health today'). The respondent should also have a copy of the EQ-5D in front of him or her for reference. If the respondent asks for clarification, the interviewer can help by re-reading the question verbatim. The interviewer should not try to offer his or her own explanation but suggest that the respondent uses his or her own interpretation.

If the respondent has difficulty regarding which box to mark, the interviewer should repeat the question verbatim and ask the respondent to answer in a way that most closely resembles his or her thoughts about his or her health today.

INTRODUCTION TO EQ-5D

(Note to interviewer: please read the following to the respondent)

We are trying to find out what you think about your health. I will first ask you some simple questions about your health TODAY. I will then ask you to rate your health on a measuring scale. I will explain what to do as I go along but please interrupt me if you do not understand something or if things are not clear to you. Please also remember that there are no right or wrong answers. We are interested here only in your personal view.

EQ-5D DESCRIPTIVE SYSTEM: INTRODUCTION

First I am going to read out some questions. Each question has a choice of five answers. Please tell me which answer best describes your health TODAY. Do not choose more than one answer in each group of questions.

(Note to interviewer: it may be necessary to remind the respondent regularly that the timeframe is TODAY. It may also be necessary to repeat the questions verbatim)

EQ-5D DESCRIPTIVE SYSTEM

MOBILITY

First I'd like to ask you about mobility. Would you say that:

1. **You have no problems in walking about?**
2. **You have slight problems in walking about?**
3. **You have moderate problems in walking about?**
4. **You have severe problems in walking about?**
5. **You are unable to walk about?**

(Note to interviewer: mark the appropriate box on the EQ-5D questionnaire)

SELF-CARE

Next I'd like to ask you about self-care. Would you say that:

1. **You have no problems washing or dressing yourself?**
2. **You have slight problems washing or dressing yourself?**
3. **You have moderate problems washing or dressing yourself?**
4. **You have severe problems washing or dressing yourself?**
5. **You are unable to wash or dress yourself?**

(Note to interviewer: mark the appropriate box on the EQ-5D questionnaire)

USUAL ACTIVITIES

Next I'd like to ask you about usual activities, for example work, study, housework, family or leisure activities. Would you say that:

1. **You have no problems doing your usual activities?**
2. **You have slight problems doing your usual activities?**
3. **You have moderate problems doing your usual activities?**
4. **You have severe problems doing your usual activities?**
5. **You are unable to do your usual activities?**

(Note to interviewer: mark the appropriate box on the EQ-5D questionnaire)

PAIN / DISCOMFORT

Next I'd like to ask you about pain or discomfort. Would you say that:

1. **You have no pain or discomfort?**
2. **You have slight pain or discomfort?**
3. **You have moderate pain or discomfort?**
4. **You have severe pain or discomfort?**
5. **You have extreme pain or discomfort?**

(Note to interviewer: mark the appropriate box on the EQ-5D questionnaire)

ANXIETY / DEPRESSION

Finally I'd like to ask you about anxiety or depression. Would you say that:

1. **You are not anxious or depressed?**
2. **You are slightly anxious or depressed?**
3. **You are moderately anxious or depressed?**
4. **You are severely anxious or depressed?**
5. **You are extremely anxious or depressed?**

(Note to interviewer: mark the appropriate box on the EQ-5D questionnaire)

EQ VAS: INTRODUCTION

(Note to interviewer: if possible, it might be useful to send a visual aid (i.e. the EQ VAS) before the telephone call so that the respondent can have this in front of him or her when completing the task)

Now, I would like to ask you to say how good or bad your health is TODAY.

I'd like you to try to picture in your mind a scale that looks a bit like a thermometer. Can you do that? The best health you can imagine is marked 100 (one hundred) at the top of the scale and the worst health you can imagine is marked 0 (zero) at the bottom.

EQ VAS: TASK

I would now like you to tell me the point on this scale where you would put your health today.

(Note to interviewer: mark the scale at the point indicating the respondent's 'health today'. Now, please write the number you marked on the scale in the box below)

THE RESPONDENT'S HEALTH TODAY

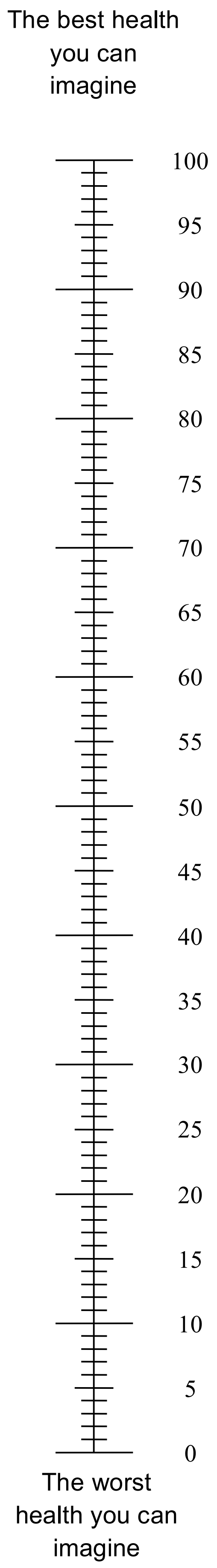

Thank you for taking the time to answer these questions.

Health Questionnaire

English version for the UK

Proxy version of the EQ-5D-5L: 1

Under each heading, please tick the ONE box that you think best describes (*insert name of person whose health is being assessed, e.g. Mr Smith's or Jane's)* health TODAY.

MOBILITY

No problems in walking about ❑

Slight problems in walking about ❑

Moderate problems in walking about ❑

Severe problems in walking about ❑

Unable to walk about ❑

SELF-CARE

No problems washing or dressing him/herself ❑

Slight problems washing or dressing him/herself ❑

Moderate problems washing or dressing him/herself ❑

Severe problems washing or dressing him/herself ❑

Unable to wash or dress him/herself ❑

USUAL ACTIVITIES *(e.g. work, study, housework, family or leisure activities)*

No problems doing his/her usual activities ❑

Slight problems doing his/her usual activities ❑

Moderate problems doing his/her usual activities ❑

Severe problems doing his/her usual activities ❑

Unable to do his/her usual activities ❑

PAIN / DISCOMFORT

No pain or discomfort ❑

Slight pain or discomfort ❑

Moderate pain or discomfort ❑

Severe pain or discomfort ❑

Extreme pain or discomfort ❑

ANXIETY / DEPRESSION

Not anxious or depressed ❑

Slightly anxious or depressed ❑

Moderately anxious or depressed ❑

Severely anxious or depressed ❑

Extremely anxious or depressed ❑

- We would like to know how good or bad you think (*insert name of person whose health is being assessed, e.g. Mr Smith's or Jane's*) health is TODAY.
- This scale is numbered from 0 to 100.
- 100 means the best health imaginable.
  0 means the worst health imaginable.
- Mark an X on the scale to indicate how good or bad you think (*insert name of person whose health is being assessed, e.g. Mr Smith's or Jane's*) health is TODAY.
- Now, please write the number you marked on the scale in the box below.

THE SUBJECT'S HEALTH TODAY [ ]

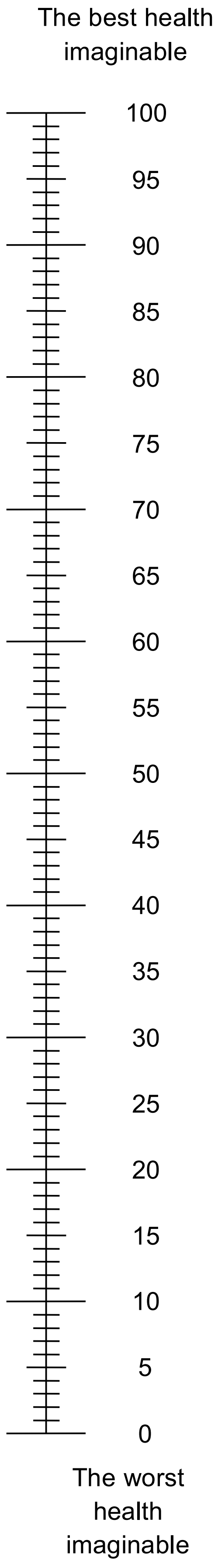

Supplement: Supplementary file 3 — Full ethically approved RIIiO pilot study protocol. (PDF 1940 kb) [file 13063_2018_3011_MOESM3_ESM.pdf]
